# Evidence of cancer-linked rodent zoonoses from biophysical genomic variations

**DOI:** 10.1038/s41598-023-41257-4

**Published:** 2023-08-26

**Authors:** Daniah Alsufyani, James Lindesay

**Affiliations:** 1 College of Sciences and Health Professions, King Saud Bin Abdulaziz University for Health Sciences, Jeddah, Saudi Arabia; 2 King Abdullah International Medical Research Center, Jeddah, Saudi Arabia; 3 Computational Physics Laboratory, Department of Physics, Howard University, 2355 Sixth Street NW, Washington, DC USA

**Keywords:** Biophysics, Physics

## Abstract

As a mechanism to explore the role of environmental adaptation in establishing the optimal distribution of single nucleotide polymophisms (SNPs) within resident homeostatic populations, relationships between quantified environmental parameters and the frequencies of the variants are being explored. We have performed sequential double-blind scans on more than 30% of chromosome 3 in an attempt to discover possible relationships using simple mathematical functions that are indicative of “adaptive forces” on the variants due to specific quantified environmental agents. We have found an association of rs13071758 with rodent zoonotic diseases. This variant is within the FHIT gene, which spans the most fragile of the common fragile sites in human lymphoblasts. FHIT, which is highly sensitive to environmental carcinogens, is partially lost in most human cancers. This finding is consistent with other studies postulating an association between rodent zoonoses and cancer. We quantify the adaptive force on the T allele as 0.28 GEUs per unit of zoonotic rodent host richness.

## Introduction

The availability of population-based data on the human genome continues to provide insight into our understanding of associations between genomic variants and environmental factors. The tools typically utilized in bioinformatics lack *universal dimensional units* that can quantify the relative degrees of various environmental pressures or stimuli upon the distribution of variants within a given population.

Some applications of statistical physics to evolutionary biology^1^ have developed a type of free energy function for the evolutionary dynamics of finite populations. In those formulations, the perturbed free energy of a population *evolves* toward fitness peaks for the allelic variants^2^. However, genodynamics^3^ does *not* focus on those survivable mutations which change crucial biological functions. Rather, it seeks to discover meaningful relationships between the distributions of genomic variants within homeostatic populations and specific environmental parameters. Given sufficient genomic data associated with an environmentally stable population, adaptive *forces* (between environments) and *powers* (within a changing or admixing population) can be quantified.

As presently formulated, genodynamics quantifies the information *dynamics* of bi-allelic single nucleotide polymorphisms (SNPs) which can be functionally related to independent variables. For this paper, the populations will be chosen to reside in ancestral geographical regions in homeostasis with the stable environments, and only *common* genomic variants will be utilized. Genodynamics differs from standard bioinformatics in the use of *universal* dynamic genomic energy units (GEUs) that allow direct comparisons between different populations. Given such (species) universal dimensional biophysical units, if mathematically smooth relationships can be established between the degree of variation of a SNP and quantified environmental parameters, the gradient of variation defines adaptive forces. Functional stability between homeostatic populations then implies that the adaptations optimize *population health*, absent any focus on disease.

## Methods

Due to ancient migrations, human populations have dispersed and successfully adapted within extreme geographic locations. As the various populations come to homeostasis within stable, survivable environments, the distribution of genomic variations within a given population will eventually be modified via enhanced transmission of more favorable traits to successive generations. Once a population reaches homeostasis within a stable environment, the maintained genomic distribution optimizes the overall health and continued survival of that population. It should be noted that some environmental pathogens (e.g., malarial mosquitos) induce variation in the genomic distribution that is beneficial for overall population health but can have adverse effects upon the health of some individuals.

Our formulation models maintained frequencies of the genomic variants of populations in multi-generational Hardy–Weinberg equilibrium within stable environments. This is done by establishing population-based stationary state variables dependent on common genomic variants. The formulation models the homeostasis of living populations made up of individuals far from *thermodynamic* equilibrium using principles motivated by thermodynamics^4–6^ to describe genomic variants within environments with persistent common agitations and stimulations.

The description of certain biological variants (like generic information content) can be modeled purely mathematically without the need of establishing universal biophysical dimensional units^7^. For instance, a dimensionless entropy ${s}^{(S)}=-\sum_{a}{p}_{a}^{(S)}{\mathrm{log}}_{2}{p}_{a}^{(S)}$ describes the degree of disorder of the alleles in a particular SNP (S) that is *not* in linkage disequilibrium as a population-averaged state variable. The overall degree of genomic disorder within a population sums over the non-linked as well as linked (haploblock) entropies, ${s}_{Genome}=\sum_{S}{s}^{(S)}+\sum_{H}{s}^{(H)}$. A relative degree of maintained *order* can be defined in terms of the normalized information content (NIC) given by

$${NIC}_{genome}=\frac{{s}_{max}- {s}_{genome}}{{s}_{max}},$$

where for bi-allelic SNPs, ${s}_{max}={N}_{SNPs}$. This dimensionless, normalized measure of the maintained order of a set of variants has values between 0 and 1, and allows direct comparisons of completely unrelated distributions. Higher NIC values are indicative of more ordered distributions. For bi-allelic SNPs, the NIC is related to *the evolutionary information density* $D\left(N\right)=1-S/N$ found in the literature^7^.

However, the establishment of universal units to describe the information *dynamics* of variation allows quantifiable comparisons of the degree of the influence of an environmental agent upon populations. A universal *dimensional* unit of $\check{\mu }=1$ GEU (genomic energy unit) has been defined as that degree of environmental agitation that invokes maximum variation (50%–50%) of the alleles in a bi-allelic SNP that is *not* in linkage disequilibrium with other SNPs. For physical systems, the external force on a system with defined potential energy is given by the gradient down the slope of the potential energy curve. When comparisons between homeostatic populations residing in differing environments generate a smooth functional dependency of a particular genomic variant upon an environmental parameter, an analogous adaptive force can be established to quantify the degree of “pressure” of that parameter upon the variant. The *adaptive force* ${f}_{a}$ of that parameter on an allele with “potential” ${\mu }_{a}$ due to environmental parameter λ will be defined as ${f}_{a}=-\frac{\partial {\mu }_{a}}{\partial \lambda }$.

A type of genomic free energy has been developed to characterize variation within populations with optimized overall health. Variations in this free energy due to the overall degree of environmental agitations is given by

$$d{F}_{Genome}=-{S}_{Genome}{dT}_{E}+\sum_{S}\sum_{a}{\mu }_{a}^{\left(S\right)}{dN}_{a}^{\left(S\right)}+\sum_{H}\sum_{h}{\mu }_{h}^{\left(H\right)}{dN}_{h}^{\left(H\right)},$$

once the allelic and haplotype potentials $${\mu }_{a}^{(S)}$$ and $${\mu }_{h}^{(H)}$$ can be quantified. In this expression, the population’s genomic entropy $${S}_{Genome}$$ is its specific genomic entropy times its size ($${S}_{Genome}=$$ $${N}_{population} {s}_{Genome})$$. The environmental potential $${T}_{E}=\frac{\check{\mu }}{{NIC}_{genome}}$$ is analogous to the thermodynamic temperature characterizing a system in thermal equilibrium, and $${NIC}_{genome}$$ quantifies the overall degree of order of the variants in the entire genome of the population.

For bi-allelic SNPs not in linkage disequilibrium, the allelic potentials satisfy

$${\mu }_{a}^{(S)}= \check{\mu }-{T}_{E}-{T}_{E}{\mathrm{log}}_{2}{p}_{a}^{\left(S\right)}.$$

These potentials are additive in genomic energy units and take the standard value of 1 GEU for maximum (50–50%) variation^4^. For the homeostatic populations explored in this paper, the environmental potentials are displayed in Table 1.

**Table 1:** The environmental potentials of the examined populations.

Population	Environmental potential
PEL	1.108
CLM	1.125
FIN	1.125
KHV	1.106
JPT	1.108
TSI	1.122
YRI	1.195
MSL	1.237

The formulation requires the calculation of the information content for bi-allelic variants of the whole genome of each population to be explored. Prior explorations with HapMap populations have demonstrated that the whole genome information content is represented to within a percent by that of chromosome 3^6^. For this reason, the information content and allelic potentials of the whole of chromosome 3 has been characterized for each of the populations explored. The populations chosen for this study were Peruvian in Lima, Peru [PEL], Colombian in Medellín, Colombia [CLM], Finnish in Finland [FIN], Kinh in Ho Chi Minh City, Vietnam [KHV], Japanese in Tokyo, Japan [JPT], Toscani in Italy [TSI], Yoruba in Ibadan, Nigeria [YRI], Mende in Sierra Leone [MSL], and Iberian populations in Spain [IBS]. Each population was scanned in a double-blind manner for a smooth functional dependency of each allelic potential on any of fifteen quantified ancestral environmental parameters including altitude, temperature, rain, bacteria, virus, protozoa, helminth, UVB, wind speed, humidity, pressure, and pathogens from chiroptera, primates, rodentia, soricomorpha. The environmental data has been population-averaged using various cities distributed in ancestral geographic regions. For non-tabular data (i.e., maps), the authors independently quantified values associated with the cities versus provided scales, and averaged results were utilized. The overall effort is expected to take more than a year to complete. In order to flag for smooth functional dependencies of allelic variations upon environmental changes, a set of a few relevant parameterized functional forms has been developed. The root-mean-squared (RMS) deviation of each allelic potential for a given population from an optimized fit of the functional form involving all populations is quantified. In an effort to emphasize the variation of allelic distributions due to environmental changes, we have chosen to only flag those smooth functional dependencies whose ratio of the RMS deviations to the maximal change in the environmental parameter (i.e., relative RMS deviation) is a dimensionless value within 10%. To this point, of the sixty thousand SNPs scanned amongst the 8 populations, only 3 have flagged using this criterion.

### Ethics approval and consent to participate

Data in this research was obtained from open access sources, and it is publicly available.

## Results

The SNP rs13071758 is only the third variant out of the first sixty thousand variants blindly examined on chromosome 3 for a simple mathematical dependency on one of the fifteen environmental parameters explored. Populations in homeostasis within environments containing agents of more robust influence display a higher degree of variation than those encountering fewer pathogens and stimulants. An established allelic potential that reflects the degree of its variation within the homeostatic population is *assumed* to optimize the overall genomic health of the population. As in our previously reported result^4^, only simple adaptive dependencies that are also monotonic for allelic potentials versus the environmental parameters are flagged. Parameterized functional forms that exhibit simple dependencies either on the frequencies of occurrence of the alleles within populations, or on the genomic potentials, are utilized. Adaptive forces are developed as the negative slope of the allelic potentials per unit change in the environmental parameters. A dataset was flagged only if the root-mean-squared deviation of the data points from the fitted curves was less than 10% of the maximum variation in the potentials. Each newly discovered adaptive force establishes an environmentally induced influence on a specific genomic variant that is likely associated with a biological function.

**Figure 1:**
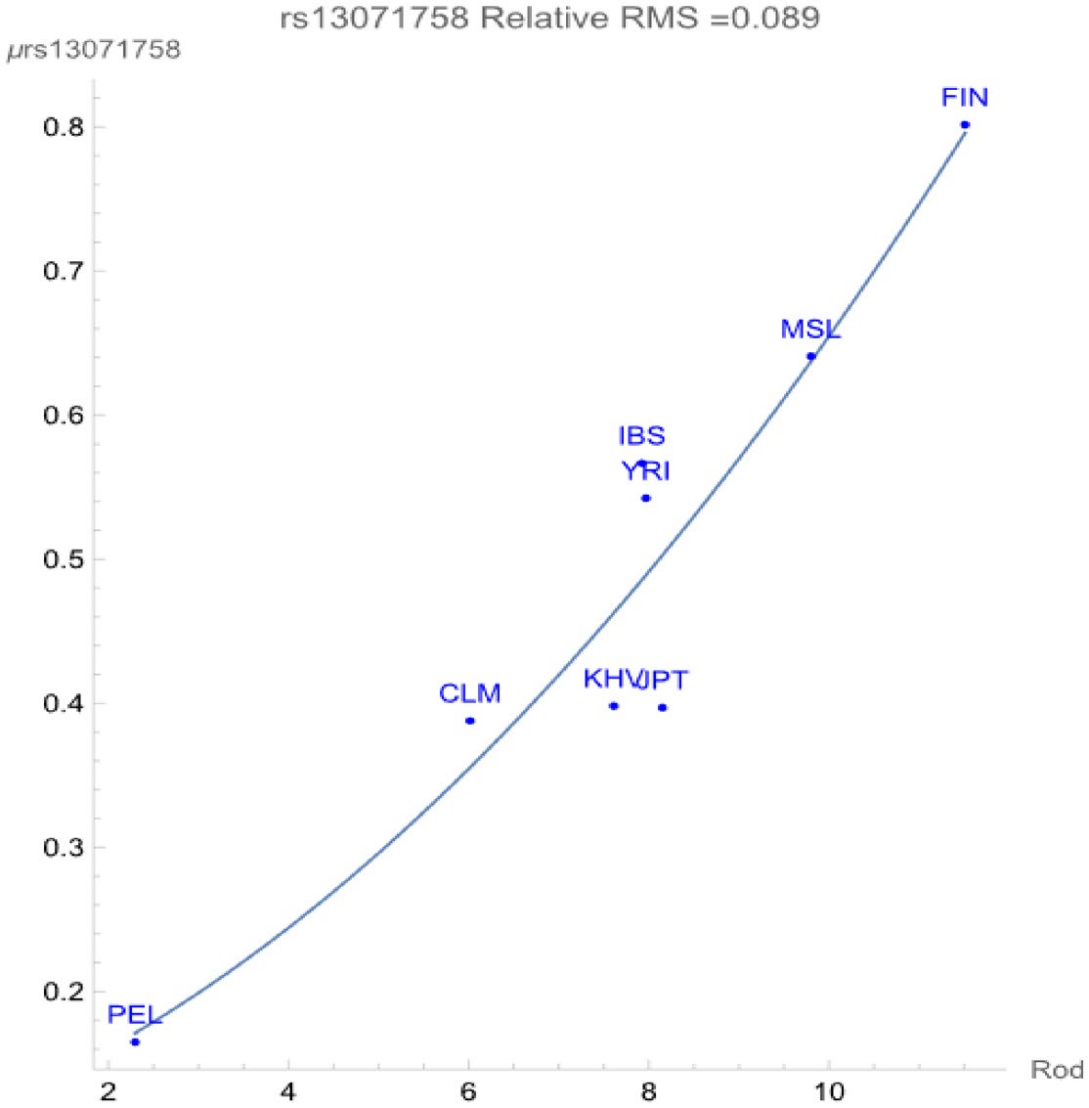
The correlation between the rs13071758 SNP potential values (in GEUs) and the richness (numbers of species) of rodent pathogens. The adaptive force is about − 0.07 GEUs/unit rodent host richness.

The variant rs13071758 only flagged a relationship between rodent zoonoses as quantified in reference^8^ versus the allelic frequencies within the eight ancestral populations as demonstrated in Fig. 1. The figure displays a direct dependency of the population averaged allelic (SNP) potential in genomic energy units versus the richness of zoonotic pathogens carried by rodents, where richness is defined in the reference as follows^8^:

"Richness: the number of unique species within a particular geographic area; richness is a count-based metric for quantifying diversity, which contrasts with other metrics, such as functional trait diversity (the different types of traits represented within a geographic area) or genetic diversity."

The SNP potential represents the population average of the two allelic potentials ${\mu }_{C}$ and ${\mu }_{T}$. The SNP flagged a relationship with a *negative* adaptive force of about − 0.07 GEUs per unit of Rodent Host Richness (RHR) within a relative root-mean-squared degree of uncertainty of about 0.089. As shown in Fig. 1, the environment with highest RHR is Finland, which also exhibits the most allelic diversity, while that with the lowest RHR is Peru, whose population exhibits the highest degree of conservation of the C allele.

**Figure 2:**
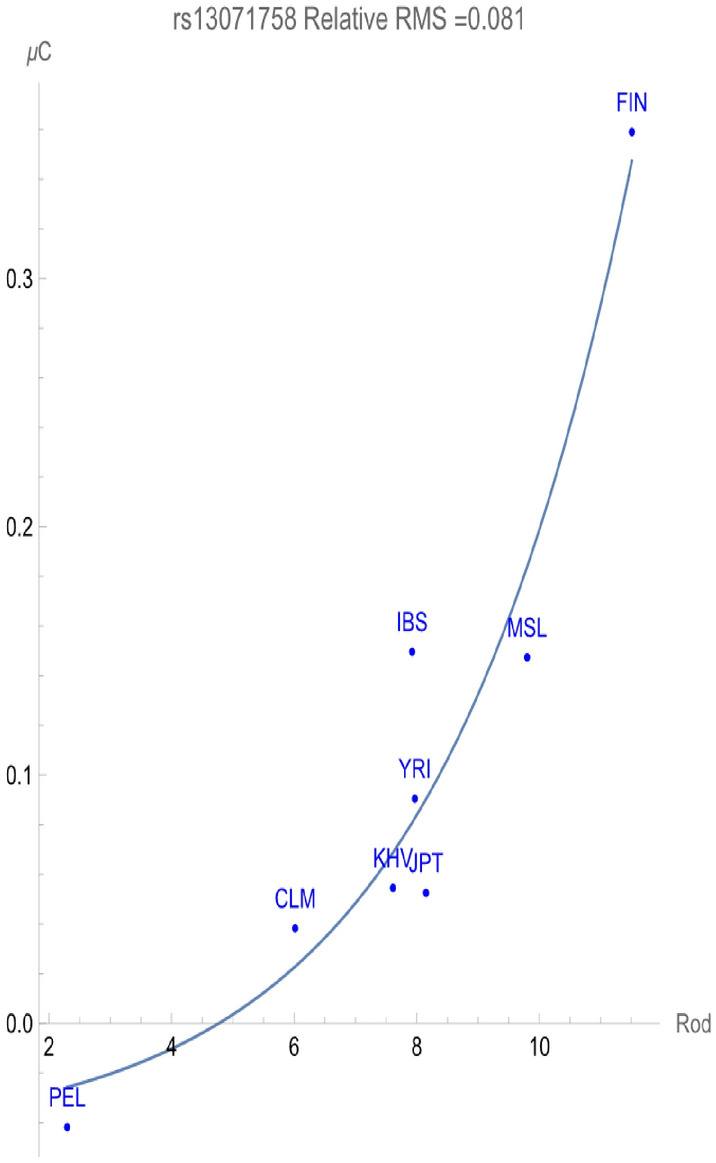
The correlation between C allelic potential (in GEUs) of rs13071758 and the rodent host richness. The adaptive force is about − 0.04 GEUs/RHR.

**Figure 3:**
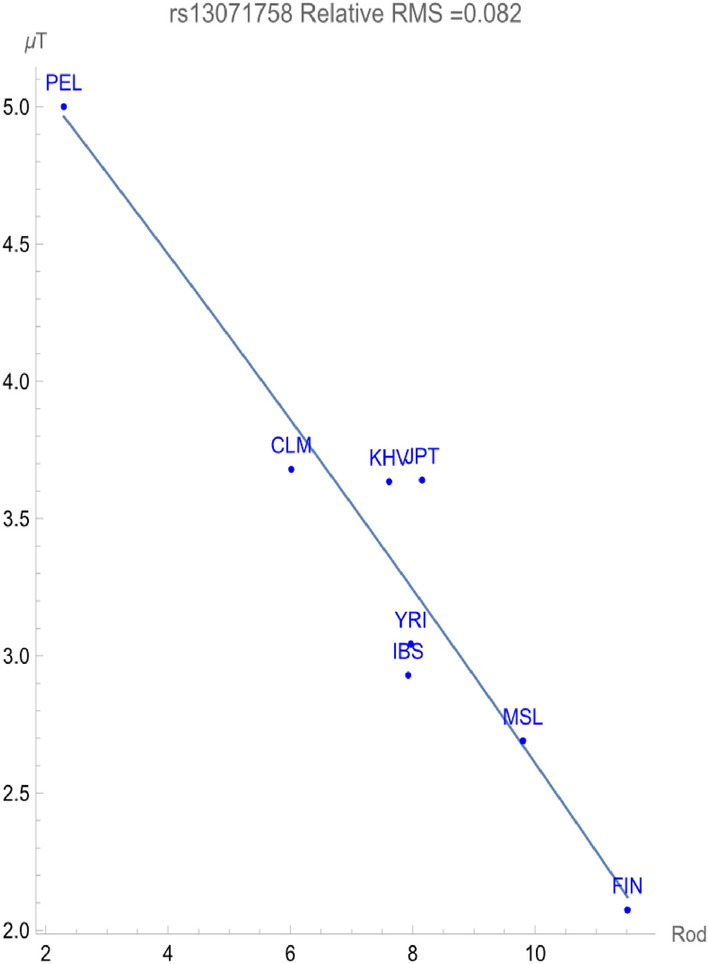
The correlation between T allelic potential (in GEUs) of rs13071758 and the rodent host richness. The adaptive force is about + 0.28 GEUs/RHR.

The frequencies of the two alleles in a bi-allelic SNP must add to one within any given population. The minor allele always has a higher allelic potential reflecting its relative rareness within members of the population. For rs13071758, the T allele is the minor allele within *all* of the homeostatic ancestral populations considered. Figure 2 demonstrates that the C allele likewise flagged for a direct relationship of allelic potential vs. RHR indicating a negative adaptive force of about − 0.04 GEUs per RHR within a relative root-mean-squared degree of uncertainty of 0.081. On the other hand, Fig. 3 exhibits that the T allele flagged a *positive* adaptive force of about + 0.28 GEUs per RHR with relative uncertainty of 0.082. This demonstrates that increased conservation of the T allele provides enhanced population health in direct relationship with increasing rodent host richness of pathogens. The adaptive force increasing the frequency of the *minor* T allele within populations is considerably larger than the others, indicating its increased significance in addressing the zoonotic rodent pathogens. However, even within the populations with highest RHR (e.g., Finland), the T allele remains the minor allele, demonstrating a significant un-flagged health benefit of the C allele. This suggests a possible genomic health benefit to the population as a whole analogous to that expressed when populations are exposed to the malarial pathogen.

## Discussion

**Figure 4:**
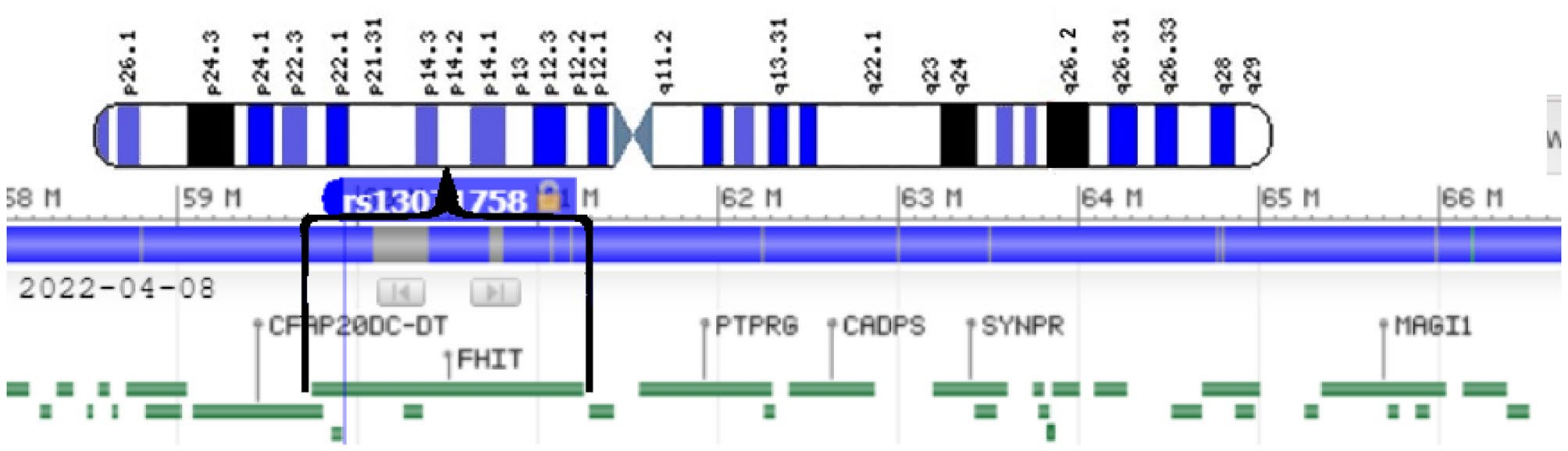
The map of the FHIT locus. Chromosome 3 has 199 million base pairs. The gene containing the flagged SNP extends from 59,747,277 to 61,251,452. The SNP rs13071758 is at locus 59,918,291.

The SNP rs13071758 (at location 59,918,291 on chromosome 3) is an intron variant of the gene Bis(5'-adenosyl)-triphosphatase, which encodes an enzyme also known as the Fragile Histidine Triad Protein (FHIT). This region of the genome has been interpreted as the human accelerated region 10, which might play a role in differentiating humans from other species^9^. The genomic region is illustrated in Fig. 4.

Common fragile sites (CFSs) on chromosomes are generally large genomic loci that exhibit chromosomal instability in response to genotoxic or replicative stress^10^. This instability (often as metaphase breaks) occurs before other genomic instabilities. When FHIT activity is absent, cells exhibit replication stress, single- and double-strand DNA breaks, and chromosomal instability. In humans, there are more than 80 common fragile sites (i.e., susceptible links of varying degrees in our chromosomes). The product of the FHIT gene is at least partially lost in most human cancers, indicating some function as a tumor suppressor. There is an indication that inactivation of only one allele in FHIT compromises its function as a tumor suppressor, suggesting that a ‘one-hit’ mechanism of tumorigensis is operative^11^.

The FHIT gene at FRA3B spans the *most* fragile of CFSs in human lymphoblasts. FHIT loss increases the fragility of other fragile sites, as well as allele copy number alterations, changes in genomic expression, and exome mutations in FHIT deficient cells. These changes *increase* the likelihood of the progression of FHIT deficient tissues to cancer. This suggests that the genomic instability within FHIT actually *regulates* global genome stability. The FHIT gene is highly sensitive to environmental carcinogens, and its function is lost within the early pre-cancerous cells of lung squamous cell carcinoma. Furthermore, the loss of FHIT has been seen to increase the fragility of other CFSs^12^. This gene is one of the earliest altered lost genes in the majority of human cancers. FHIT has roles in apoptosis and prevention of the epithelial-mesenchymal transition. Since the FHIT gene is not the *most* fragile locus in epithelial cells (the cells of origin for most FHIT deficient cancers), it is suggestive that the deletions are not just being replicated as cancer due to DNA damage, but primarily due to increased vulnerability to DNA damage due to the loss of the FHIT function^13^.

The FHIT gene is a member of the histidine triad gene family involved in purine metabolism. Purines are basic components of nucleotides in cell proliferation, and thus impaired purine metabolism is associated with the progression of cancer. Although the exact molecular function of the protein encoded by this gene remains partially unclear, the gene has also been demonstrated in animal studies to work as a tumor suppressor^14–16^. FHIT has been shown to synergize with the tumor suppressor VHL in protecting against chemically-induced lung cancer^17^.

Further evidence supports that FHIT is a genomic ‘caretaker’ whose loss initiates genome instability and the onset of cancer development in humans. FHIT loss brings on replication stress-induced chromosomal alterations in cells absent any DNA damage response (in contrast to oncogene-activated cells). It has been found that potentially pre-cancerous mutated genes (oncogenes) that are abnormally expressed (ectopic) in mice induce clonal tumors rather than systemic tumors that affect the entire body^18^. However, in human cancers the oncogenes are *not* ectopically activated, but only activate in tumor cells. FHIT deficient mice have been found to develop normally and live long lives, which implies that FHIT inactivation can initiate genome instability without compromising fitness at the cellular and organism levels^13^.

Our results flagged an adaptive force favoring increased conservation of the T allele of the intron variant rs13071758 in FHIT correlated with increasing zoonotic host richness and diversity arising from rodents^8^. FHIT was the first fragile gene with a developed and characterized mouse knockout model^16^. FHIT knockout mice are much more susceptible to cancer induction than wild-type mice. This chromosomal fragile site is susceptible to DNA gaps and breaks on exposure to carcinogens in both mice and humans^15^.

Animal viruses and bacteria are ubiquitous in the environment, but little is known about their transmission to humans, or what role any might play in human cancer. For instance, certain viruses can interfere with the host’s immune system to cause cancer without integrating into the victim’s DNA. Furthermore, it is known that animal viruses potentially express oncoproteins (encoded by the oncogenes associated with the growth of cancer tumor cells) in human cells, despite the more stringent replicant restrictions in humans^19^. It should be noted that FHIT *is* involved in replicate restrictions.

Farm and domestic animals that feed on or share habitat with infected rodents might indirectly expose humans to rodent-borne pathogens. For instance, in the case of orthopox infection, cats actively transmit the pathogen from rodents to humans. Exposure to respiratory or urinary aerosols from rodents poses risk for various human infections (e.g., Hantavirus, Mycoplasma, pulmonis, Streptococcus pneumoiae, Staphylococcus aureus, and Pasteurella multocida)^20^. Conversely, human polyomaviruses have been found to cause tumors in rodents. KIPvV and WUPyV polyomaviruses have been detected in children with lower respiratory tract disease^21^.

Studies have found that a significant fraction of all cancers (~ 20%) have been associated with co-infections involving microbes^22–25^. This suggests a role of inflammation and immune response in cancer development^26^. An examination of lung cancer risk amongst patients with pneumococcal pneumonia found an association of increased risk by a factor of 3.25 versus controls with a 95% confidence interval. The hazard ratio increased to 4.24 after adjustment for age, gender, and co-morbidities^27^. Another study found an increased risk of 7 specific cancers (cervical cancer, multiple myeloma, leukemia, sarcoma, liver cancer, pancreatic cancer, and urinary tract cancer) during the first year after hospitalization for *Staphylococcus aureus* Bacteremia. During the first year of follow up, the number of cases versus controls within the study that were diagnosed with incident cancer corresponded to a 65% increased risk. After the first year, the overall risk of cancer was comparable in the case and population cohorts^26^. Finally, the Pasteurella multocida toxin is a highly potent mitogen, inducing or enhancing the rate of cell division. Furthermore, it has been demonstrated to block apoptosis, as well as activating heterotrimeric G-proteins which have potential roles in carcinogenesis^28^.

The mouse mammary tumor virus (MMTV) is well established as an etiologic agent of mammary tumors in mice^29^. MMTV can infect and multiply in human breast epithelial cancer cell lines^30^, and its gene sequence co-exists in many human breast cancers^29^. There is compelling evidence that MMTV likely has a causal role in up to 40% of human breast cancers, and its life cycle in humans is almost the same as in mice^31^. This virus likely also causes biliary cirrhosis (bile duct cancer)^31^. With regards to the ease by which this virus can be transmitted, MMTV gene sequences are present in the saliva of 27% of healthy children, 11% of healthy adults, and 57% of female breast cancer patients^32^.

## Data Availability

The datasets generated and/or analysed during the current study are available in Ensembl repository, Ensembl genome browser 108 and in Science Direct repository, Global Patterns of Zoonotic Disease in Mammals—ScienceDirect.
